# Supplementary material for: Patterns of Genital Tract Mustelid Gammaherpesvirus 1 (Musghv-1) Reactivation Are Linked to Stressors in European Badgers (Meles Meles)
Source: Biomolecules. 2021 May 11;11(5):716. doi: 10.3390/biom11050716 (PMC8151406; doi:10.3390/biom11050716)
Supplement: Supplementary file 1 [file biomolecules-11-00716-s001.zip › biomolecules-1192504-supplementary.pdf]

biomolecules

Supplementary materials

Patterns of Genital Tract *Mustelid Gammaherpesvirus 1* (Musghv-1) Reactivation Are Linked to Stressors in European Badgers (*Meles Meles*)

Ming-shan Tsai [1,*], Sarah François [2], Chris Newman [1,3], David W. Macdonald [1] and Christina D. Buesching [3,4]

1 Recanati-Kaplan Centre, Wildlife Conservation Research Unit, Department of Zoology, University of Oxford, Abingdon Road, Tubney House, Tubney, Oxfordshire OX13 5QL, UK; chris.newman@lmh.ox.ac.uk (C.N.); david.macdonald@zoo.ox.ac.uk (D.W.M.)
2 Evolve.Zoo, Peter Medawar Building for Pathogen Research, Department of Zoology, University of Oxford, South Park Road, Oxford OX1 3SY, UK; sarah.francois@zoo.ox.ac.uk
3 Cook's Lake Farming Forestry and Wildlife Inc (Ecological Consultancy), Queens County, NS B0J 2H0, Canada; christina.buesching@ubc.ca
4 Department of Biology, Irving K. Barber Faculty of Science, University of British Columbia, Kelowna, BC V1V 1V7, Canada
* Correspondence: mingshan.tsai@zoo.ox.ac.uk

Citation: Tsai, M.-s.; François, S.; Newman, C.; Macdonald, D.W.; Buesching, C.D. Patterns of Genital Tract *Mustelid Gammaherpesvirus 1* (Musghv-1) Reactivation Are Linked to Stressors in European Badgers (*Meles Meles*). *Biomolecules* **2021**, *11*, 716. https://doi.org/10.3390/biom11050716

Academic Editor: Marshall Williams

Received: 6 April 2021
Accepted: 6 May 2021
Published: 11 May 2021

Publisher's Note: MDPI stays neutral with regard to jurisdictional claims in published maps and institutional affiliations.

Copyright: © 2021 by the authors. Licensee MDPI, Basel, Switzerland. This article is an open access article distributed under the terms and conditions of the Creative Commons Attribution (CC BY) license (http://creativecommons.org/licenses/by/4.0/).

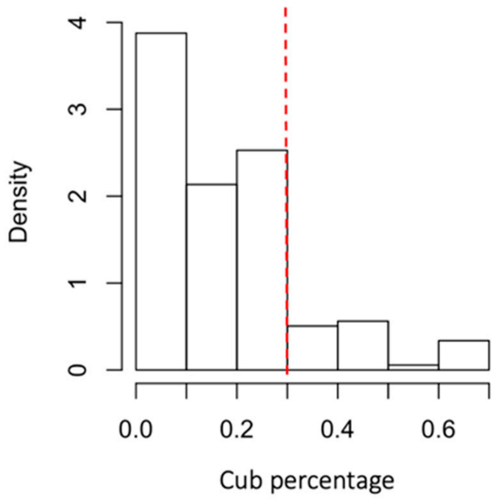

Figure S1. Data distribution of cub percentage (n=251) according to group density. The cutoff point of 30% (red dashed line) is used to divide the tail from the distribution (left of the line).

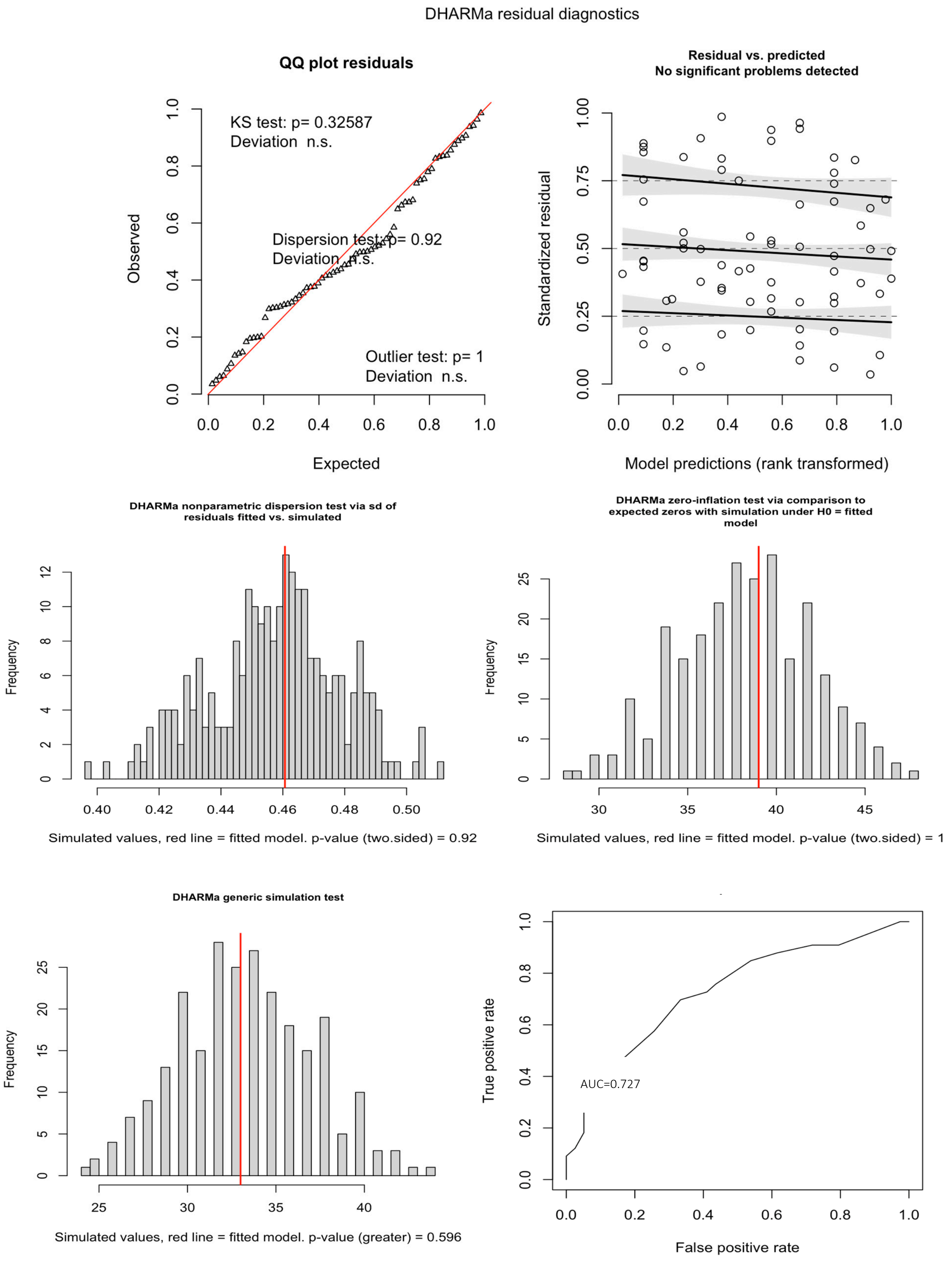

Figure S2. Residual diagnostic plot of the final multivariable logistic regression model of juvenile MusGHV-1 shedding in genital tract.

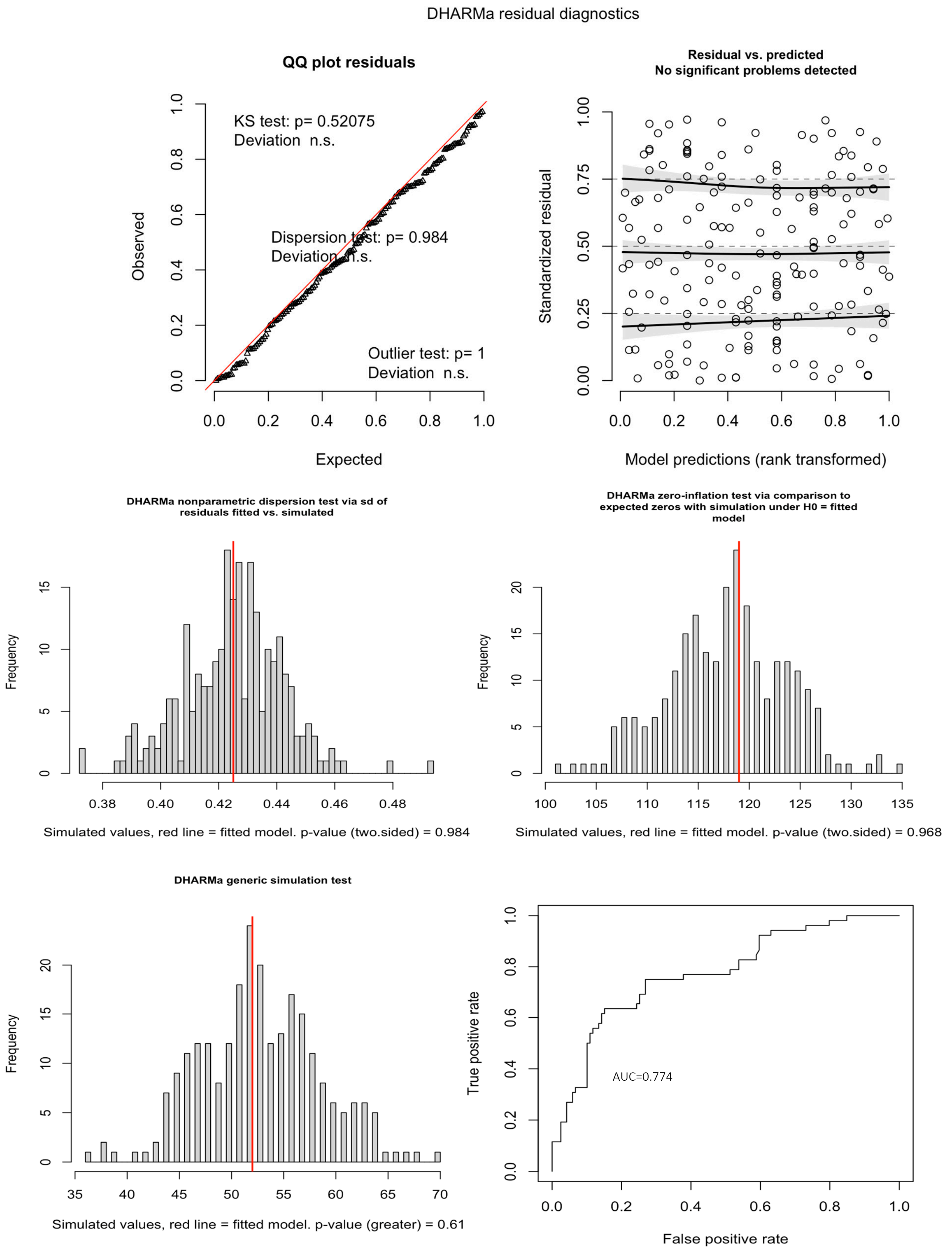

Figure S3. Residual diagnostic plot of the final multivariable logistic regression model of adult MusGHV-1 shedding in genital tract.
